# Recent Cutting‐Edge Technologies for the Delivery of Peptide Nucleic Acid

**DOI:** 10.1002/chem.202500469

**Published:** 2025-05-21

**Authors:** Concetta Avitabile, Maria Teresa Cerasa, Antonia D'Aniello, Michele Saviano, Maria Moccia

**Affiliations:** ^1^ Consiglio Nazionale delle Ricerche Istituto di Cristallografia URT‐Caserta via Vivaldi 43 Caserta 81100 Italy; ^2^ Dipartimento di Scienze e Tecnologie Chimiche Università di Roma Tor Vergata via della Ricerca scientifica 1 Roma 00133 Italy; ^3^ Consiglio Nazionale delle Ricerche Istituto di Cristallografia Strada Provinciale 35d, n. 9, Montelibretti (RM) Caserta 00010 Italy

**Keywords:** calixarene, delivery systems, liposomes, nanoparticles, peptides

## Abstract

Peptide nucleic acids (PNAs) have garnered significant attention due to their enhanced chemical, physical, and binding properties in comparison to natural nucleic acids. This prompted their application in antigene/antisense approach, assigning them a pivotal role in gene editing and, more recently, showing their potential as “bilingual” molecules being able “to speak” both nucleic acid and protein language. However, to expand the applications of PNAs in therapy, the challenge of effectively delivering PNAs to cells needs to be addressed. Among several delivery approaches employed so far, the nanotechnology‐based ones showed great potential. In this review, we provide an overview of the latest in the field (2019 to present), beginning from peptide‐based delivery systems, as well as cutting‐edge approaches involving nanoparticles, liposomes, and calixarene, showing how they have inspired the development of smarter delivery approaches to boost PNAs applications.

## Graphical abstract

This review provides an overview of PNA cellular delivery methods, starting with traditional peptide‐based systems and progressing to advanced approaches using nanoparticles, liposomes, and calixarene. It highlights how these innovative strategies have opened the way for more effective and efficient PNA delivery, ultimately enhancing the potential for broader therapeutic applications of PNAs.

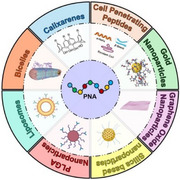

## 1 Introduction

**Figure 1:**
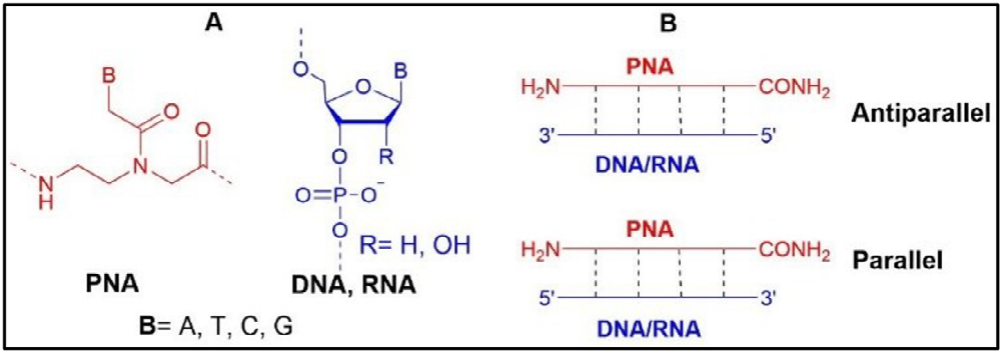
a) Chemical structures of PNA, DNA, and RNA, b) PNA‐DNA/RNA recognition mode.

Peptide Nucleic Acid (PNA) is a synthetic DNA/RNA mimic composed of a pseudopeptide backbone made of N‐(2‐aminoethyl) glycine (*aeg*) motifs, replacing the sugar‐phosphate backbone of natural nucleic acids (Figure 1a).^[^ ^1^ ^]^ Nucleobases are attached to the main skeleton via a carbonyl methylene linker. Despite these fundamental structural differences, PNAs are still capable of forming Watson–Crick hydrogen bonds. PNAs exhibit stronger binding affinity to complementary DNA/RNA oligomers, increased specificity, and excellent single‐nucleotide mismatch discrimination.^[^ ^2^, ^3^, ^4^ ^]^ Due to these properties, PNAs can target structured DNA and RNA in a sequence‐specific manner through various mechanisms of action (Figure 1b).^[^ ^5^ ^]^ This is a key aspect of nucleic acid targeting, as DNA and RNA adopt various structured forms under physiological conditions, making them less accessible. PNA sequences are resistant to nuclease‐ and protease‐mediated degradation in serum and cell extracts, which extends their lifetime both in vitro and in vivo making them an ideal potential sequences for therapeutic and diagnostic applications.^[^ ^6^ ^]^ Unlike DNA, PNAs remain stable across a wide range of temperatures and pH levels.^[^ ^7^ ^]^

Since their discovery, PNAs have gained considerable attention across various research fields due to their potential applications in diagnostics and pharmaceutics. PNAs represent an outstanding molecular tool that can be used for different applications, including a) controlling gene expression through antisense,^[^ ^8^ ^]^ antigene^[^ ^9^ ^]^ approaches, or inducing gene editing^[^ ^10^ ^]^; b) acting as anti‐infective agents, including antimicrobial and antibacterial properties;^[^ ^11^ ^]^ c) developing advanced diagnostics tools and biosensors with enhanced recognition capabilities;^[^ ^12^ ^]^ d) targeting different class of RNA such as miRNA, siRNA, and lnRNA etc., for both diagnostic^[^ ^13^ ^]^ and therapeutic purposes;^[^ ^14^, ^15^ ^]^ f) supporting recombinant processes with exogenous DNA targets to enable genome editing or repair using PNAs, especially those capable of forming triplex structures with target DNA, like bis‐PNAs and tail‐clamp PNAs;^[^ ^16^ ^]^ Recently, PNA‐based therapeutics have reached preclinical and clinical development, with examples such as OLP‐1002 (for osteoarthritis) and NT‐0200 (for myotonic dystrophy), showcasing promising results in modulating gene expression via pre‐mRNA binding with high selectivity and safety profiles;^[^ ^16^ ^]^ g) serving as smart materials that combine peptide backbones and nucleobases, allowing them to self‐assemble and create complex structures. This multi‐component arrangement can generate various supramolecular structures due to their unique chemical and physical properties.^[^ ^17^ ^]^

Despite the outstanding properties of PNA,^[^ ^18^ ^]^ significant challenges still need to be addressed before its clinical application. PNA oligomers suffer from poor water solubility, due to the neutrality of their backbone, which makes them prone to aggregation.^[^ ^19^ ^]^ Poor water solubility also results in a low cellular uptake. Without passing through the cell membrane, PNAs cannot interact with target sequences and therefore the biological activity cannot be exerted. Some modifications, such as the addition of positively charged residues at the terminals or conjugation to negatively charged molecules like DNA or carrier peptides, have been introduced to improve solubility and cellular uptake. While PNAs have demonstrated antisense and antigene effects in cell‐free systems, further investigations into their potential as gene therapeutic drugs have been hampered by the poor intrinsic uptake of PNA in living cells.^[^ ^20^ ^]^ The antisense action of PNAs occurs through steric blockage of the corresponding mRNA,^[^ ^21^, ^22^ ^]^ without any enzymatic digestion, as observed in the RNAse‐H or RNA‐Induced Silencing Complex (RISC)‐mediated cleavage of other antisense oligonucleotides and siRNA. Similarly, PNA‐based antigene or anti‐miRNA systems rely solely on tight binding to their targets. Therefore, maximizing both their binding ability and intracellular concentration is crucial for achieving a therapeutic effect. Considering great therapeutic and diagnostic potential of PNAs, it has been, and remains, of primary importance to develop new and efficient delivery approaches to further enhance their potency and preclinical development. In addition to their use as antisense and antigene agents, PNAs have also emerged as promising tools in gene editing, where their ability to invade double‐stranded DNA and recruit endogenous repair mechanisms—without the need for nucleases—offers an innovative and potentially safer alternative to CRISPR‐Cas systems.^[^ ^16^ ^]^ To improve cellular uptake by promoting membrane association/endocytosis, PNAs must be conjugated, formulated, or modified in the backbone.^[^ ^23^ ^]^ Given the wide range of PNA applications, there is an urgent need to develop new and more efficient delivery systems to expand their therapeutic potential. We herein report an overview of the most recent advances in the field, highlighting the most effective approaches (peptide‐based delivery systems, also characterized by chemical modified PNAs, designed to enhance cellular uptake) to forefront nanotechnologies: nanoparticle‐based delivery systems, liposomes, calixarene, etc. showing how they inspired the development of smarter delivery approaches to boost their applications.

## 2 Challenges in PNA Cellular Uptake

**Figure 2:**
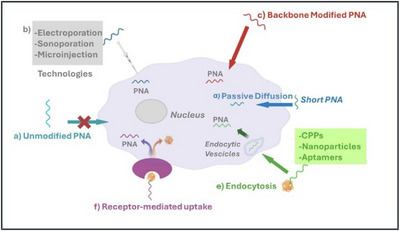
Method for the uptake of PNA into the cell: a) unmodified PNA; b) technologies for PNA delivery (electroporation, sonoporation, microinjection); c) backbone‐modified PNA; d) passive diffusion; e) endocytosis; f) receptor‐mediated uptake.

PNAs struggle to cross lipid membranes due to their uncharged backbone (Figure 2a), limiting their biological applications. Various technologies have been developed to improve cellular uptake, including electroporation,^[^ ^24^ ^]^ sonoporation,^[^ ^25^ ^]^ and microinjection.^[^ ^26^ ^]^ Additionally, modifications involving cationic transfection reagents^[^ ^27^ ^]^ and conjugation to small and large molecules such as lipophilic moieties, sugar, polymer nanoparticles, cell‐penetrating peptides (CPPs), chemically modified PNAs have been employed to enhance internalization (Figure 2).

### 2.1 Physical Delivery Methods

The most widely used nonviral physical transfection methods for PNA delivery include electroporation,^[^ ^24^, ^25^ ^]^ sonoporation and microinjection (Figure 2b).^[^ ^26^ ^]^ Electroporation involves the application of electrical pulses to cells, creating transient pores in the cell membrane, allowing PNAs to enter; sonoporation uses ultrasound waves to induce temporary disruptions in the cell membrane, allowing PNAs to pass through; microinjection directly injects “naked” PNA sequences into individual cells via a fine needle, an approach unsuitable for large‐scale or in vivo applications due to its invasiveness.

### 2.2 Cellular Uptake Mechanisms

PNAs can enter cells through various mechanisms, depending on the employed delivery method. Common pathways include passive diffusion, endocytosis, receptor‐mediated uptake, and the use of CPPs (Figure 2d‐f).

Small‐sized PNAs can passively diffuse into cells, driven by concentration gradients (Figure 2d). This process occurs through the hydrophobic core of the lipid bilayer. However, passive diffusion is generally inefficient and is more effective for shorter PNAs with lower molecular weights.

Many PNA delivery systems rely on endocytosis, a process in which cells engulf extracellular molecules by forming vesicles around them. When PNAs are conjugated to molecules or particles that facilitate endocytosis, such as CPPs, nanoparticles, or aptamers, they are internalized by cells through these endocytic pathways. Once inside the cell, the PNAs, contained within endocytic vesicles, undergo intracellular trafficking and often fuse with endosomes or lysosomes (Figure 2e). For the PNAs to exert their effects in the cytoplasm or nucleus, they must escape from these vesicles. In some cases, PNAs can also enter cells via receptor‐mediated endocytosis (Figure 2f), a process driven by specific interactions between ligands on the surface of PNAs and receptors on the cell surface. When PNAs are conjugated to ligands that bind to these receptors, they are recognized and internalized by the cells through endocytic pathways. This mechanism provides a targeted delivery approach, allowing PNAs to selectively enter cells that express the corresponding receptors.

CPPs are small peptides that cross the cell membrane and carry cargo molecules into the cytoplasm or nucleus. When PNAs are conjugated to CPPs, the resulting CPP‐PNA conjugates interact with the cell membrane and are taken up by the cell via mechanisms such as direct translocation or endocytosis. Once inside the cell, CPP‐PNA conjugates can exert their biological effects in the cytoplasm or nucleus. However, delivering PNA into the cytosol and nucleus remains challenging, as cell membranes have a negative charge and cationic transfection reagents are required to co‐transfect the PNA/DNA complexes.^[^ ^28^, ^29^, ^30^ ^]^ Although high concentrations of CPP‐PNA conjugates can trigger endocytosis, they often remain trapped within the endosomes. Strategies such as adding calcium ions, chloroquine, or sucrose can facilitate the release of PNAs from endosomes in cell culture; however, these approaches are not feasible for clinical application.^[^ ^31^, ^32^ ^]^

### 2.3 Cellular Delivery Through Backbone‐Modified PNA Structures

Several modifications have been proposed to the PNA backbone to enhance cellular permeability, as standard neutral PNA sequences cannot spontaneously enter cells. These modifications highlight the importance of balancing PNA structure with its functional effectiveness. While improving cellular uptake, it is crucial to preserve key properties such as binding specificity and stability. The primary strategy has involved introducing partial charges into PNA oligomers to facilitate their passage across the lipid bilayer.^[^ ^33^ ^]^

**Figure 3:**
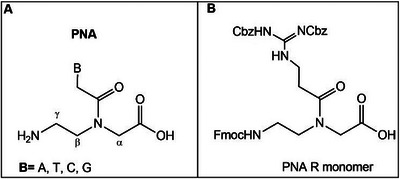
a) PNA‐modified backbone; b) PNA R monomer.

These modifications, which include the introduction of anionic and cationic substituents as well as alterations to the pseudopeptide backbone, have shown promising results.^[^ ^34^ ^]^ The modifications can occur on the backbone, on the carbonyl methylene bridge, and on the nucleobases (Figure 3A). Here, we will describe only the backbone modifications, as these have yielded the best results not only in terms of cellular uptake but also in efficiency and selectivity.

#### 2.3.1 Guanidinium Backbone Modifications

GPNA (Guanidinium PNA) was one of the first modifications to the standard *aeg*PNA. Argininic fragments were incorporated in α or γ position of the backbone, creating an amphiphilic oligo structure thus increasing permeability.^[^ ^35^ ^]^ To compare the delivery ability of GPNA, three systems were conjugated to fluorescein: *aeg*PNA, GPNA, and transactivating transcriptional activator (TAT), a well‐known peptide carrier. The study was carried out employing both colon (HCT116) and osteosarcoma cells, which revealed that GPNA crossed the membrane with the same efficiency as the TAT complex. Both systems were localized in the nucleus.^[^ ^35^ ^]^ Several studies have shown that the modification in the γ position of PNA oligomers improves cellular permeability, reduces aggregation, and enhances affinity and selectivity for the target. The introduction of a functional group at the γ position provides better structural pre‐organization, which boosts the interaction between PNA oligomers and their target molecules.^[^ ^36^ ^]^ These findings suggest that the modification at the γ position may be more effective than α modifications. Krishna and coworkers^[^ ^37^ ^]^ reported a guanidinium (protonated form of guanidine)‐based PNA monomer, named R, to improve recognition and bioactivity and therefore improve cellular availability (Figure 3B). The R‐monomer, included in the PNA sequences named nucleobase‐modified dsRNA‐binding PNAs (dbPNAs), was able to target dsRNAs. The preliminary study was carried out on *Spodoptera frugiperda* (Sf 9) insect cells, which were incubated with labeled‐modified PNAs for 12 hours. The R‐modified PNAs displayed fluorescence signals predominantly in the cytoplasm, whereas *Sf9* cells treated with unmodified PNA exhibited no significant signals. This indicates that the introduction of R residues enhanced the cellular delivery.^[^ ^37^ ^]^

**Figure 4:**
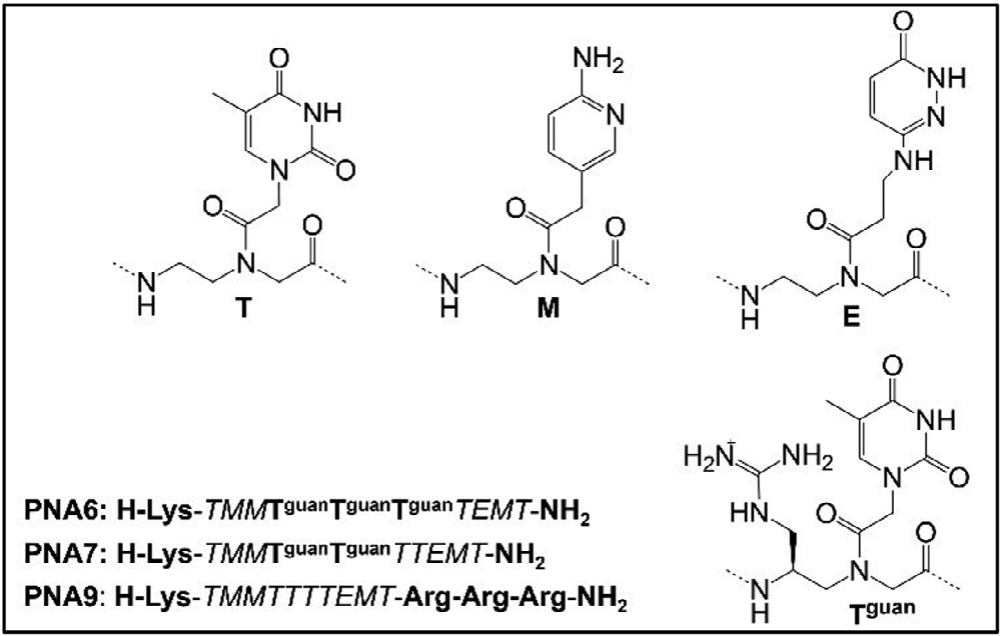
Sequence of PNA1, PNA 6, PNA 7, and PNA 9.

An alternative backbone modification, modified γ‐(S)‐guanidylmethyl PNAs, was proposed by Virta and coworkers.^[^ ^38^ ^]^ The study investigated the internalization of four different modified PNAs with three or two consecutive γ‐(S)‐guanidylmethyl groups named, respectively, PNA 6 and PNA 7, one with 3 argininic units, PNA 9, and an aminopyridine‐modified PNA, PNA 1 (Figure 4). These four PNAs were conjugated at N‐terminus with HF488 (HiLyte Fluor 488) and tested in PC‐3 prostatic cancer cells. Even at low concentration (5 µmol L^−1^), PNA 7, PNA 6, and PNA 9 exhibited distinct fluorescent signals within the cytoplasm. The intensity of fluorescence decreased progressively among these three, while PNA 1 was only able to penetrate the cellular membrane at higher concentration (10 µmol L^−1^). This study shows that γ‐(S)‐guanidylmethyl modifications improve PNA cellular delivery, although they are less effective than arginine conjugates. Both modifications include a guanidine headgroup, which is essential for the efficient cellular uptake of arginine‐based oligomers compared to other polycation sequences. Mitra and Ganesh strategically incorporated an aminomethylene group at the C2 and C5 positions (α/γ‐amPNA). The two cationic modified PNA sequences were labeled with fluorescein and compared to unmodified PNA for uptake experiments in HeLa cells. The results showed γ‐am PNA could enter cells through an effective energy‐dependent mechanism of internalization.^[^ ^39^ ^]^

#### 2.3.2 Novel Backbone Architecture

**Figure 5:**
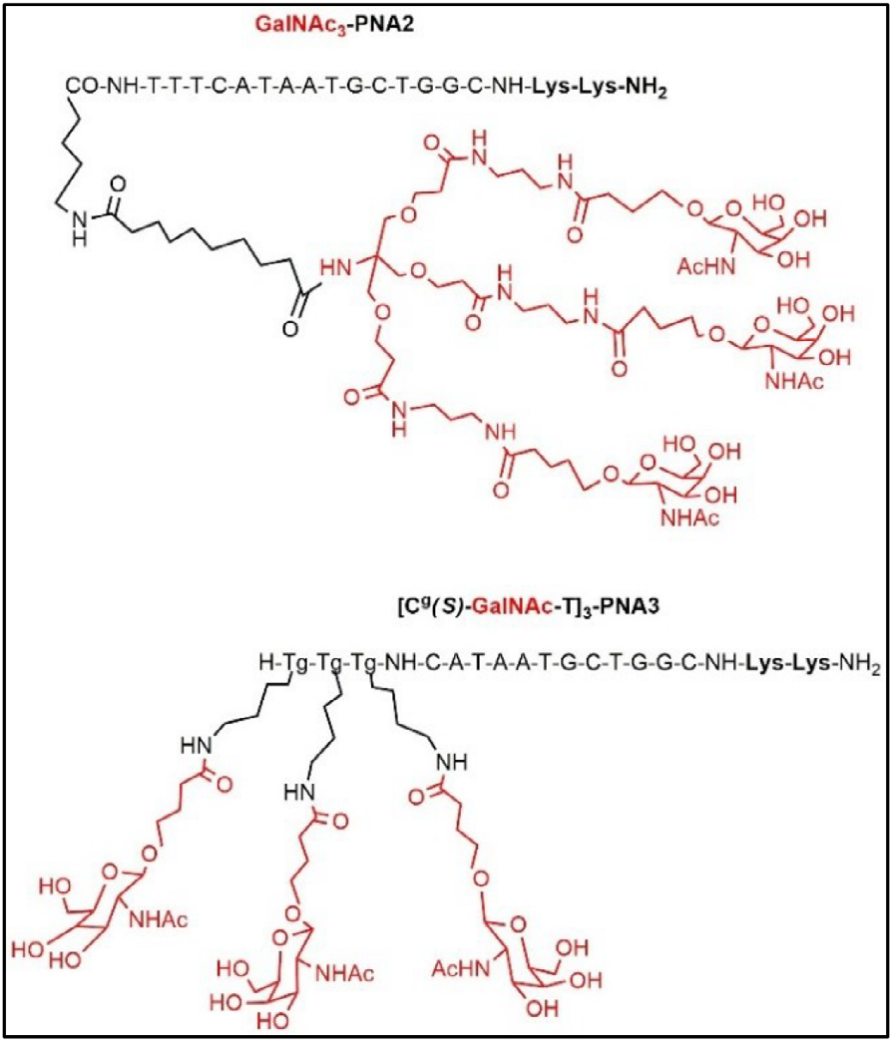
Sequence of GalNAc3‐PNA 2 and [Cγ(S)‐GalNAc‐T]3‐PNA 3; structures adapted from ref. [38, 40].

An alternative approach to improve cellular uptake of PNA is through the conjugation of different glycans. Bhingardeve et al.^[^ ^40^ ^]^ proposed the use of PNA conjugated with N‐acetylgalactosamine (GalNAc) in two distinct approaches: (i) a triantennary branched structure linked at the N‐terminus of the PNA through a spacer chain and (ii) a sequential architecture wherein three monovalent GalNAc units were attached to the Cγs of the backbone at three successive N‐terminal PNA residues (Figure 5). Both types of GalNAc‐conjugated PNAs formed stable duplexes with complementary DNA. The comparative study involved six different sequences: the traditional *aeg*‐PNA, the N‐terminal GalNAc_3_‐PNA2, a linear PNA with three sequential GalNAc units a Cγ‐site ([Cγ(S)‐GalNAc‐T]3‐PNA 3), and their corresponding fluorescent derivatives.

Uptake studies were performed on hepatocyte cells (HepG2) that express a sialo‐glycoprotein receptor (ASGPR) recognizing GalNAc ligands. The results showed the highest permeability for [Cγ(S)‐GalNAc‐T]3‐PNA 3 (about 40 times more efficient than PNA1) and good cellular internalization for GalNAc_3_‐PNA2 (13 times higher than *aeg*‐PNA). The promising results from GalNAc_3_‐modified PNAs in liver cells indicate potential for targeted therapies, although they raise concerns about the effectiveness in other cell types.

**Figure 6:**
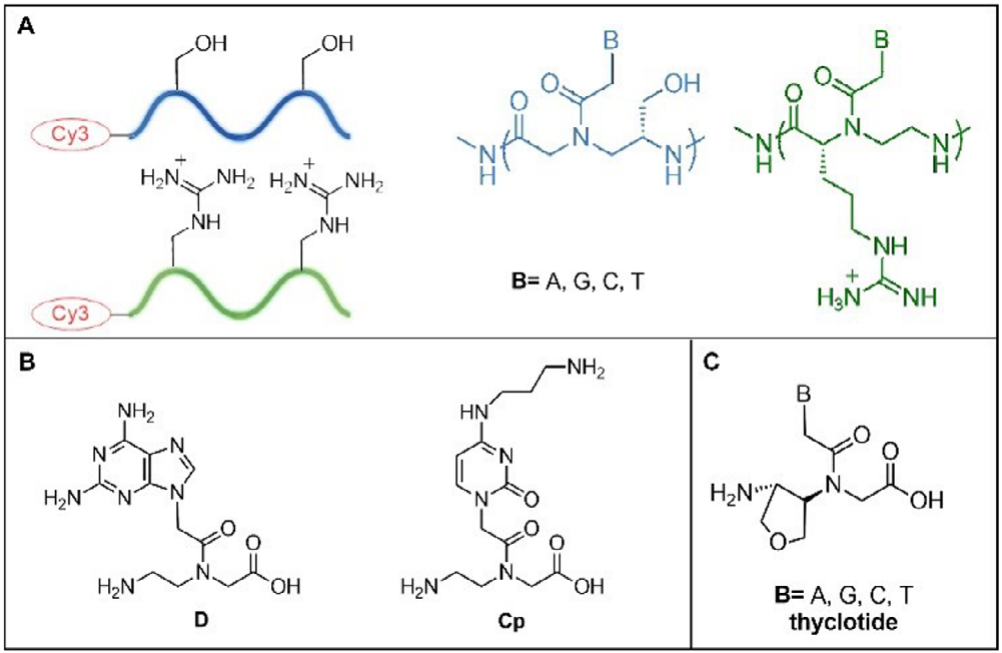
a) Ser‐PNA and Arg‐PNA; b) D‐monomer and Cp‐monomer; c) thyclotide.

Winssinger and colleagues^[^ ^41^ ^]^ conducted an in‐depth study involving two sets of modified PNA sequences. One set consisted of PNAs with serine side chains at the γ‐position, while the other set featured Arg‐PNAs with a guanidinium group at the third position (Figure 6A). Initially, the researchers observed that the Arg‐PNAs were more efficient at entering cells compared to the Ser‐PNAs. However, they made an interesting finding when they introduced a protein called Caprin‐1 into the supernatant of HeLa cells.

After 3 hours of incubation, they noticed that the uptake of Serine‐PNAs by the cells had significantly increased, while the uptake of Arg‐PNAs remained unaffected. Moreover, when Caprin‐1 was directly mixed with the PNAs, the Arg‐PNAs showed a more pronounced increase in cellular uptake compared to the Ser‐PNAs. This suggested that Caprin‐1 plays a role in enhancing PNA uptake, with a stronger effect on the Arg‐modified PNAs. This discovery highlights the potential for certain proteins, such as Caprin‐1, to facilitate the delivery of PNAs into cells. It also suggests that the effectiveness of PNA delivery may be influenced by the presence of specific proteins in different cell types, which could help tailor and improve PNA‐based therapies. A simple modification to the traditional backbone of PNA^[^ ^42^ ^]^ was proposed by Falah and his group. They designed three PNA‐based strands: PNA 1, with three lysine residues at the N‐terminal; PNA 2, an *aeg*‐PNA incorporating three lysine residues and a D monomer (a PNA‐A monomer with an additional amino group in position 2); and PNA 3, where the C monomer was replaced with a Cp monomer, containing an aminopropyl units (Figure 6B). These PNAs were synthesized to target the mRNA of the *KRAS* gene, which is crucial in pancreatic cancer pathways. To evaluate cellular uptake, PNA 1 was labeled with fluorescein isothiocyanate (FITC), resulting in fluorescent signals within the cytoplasm. Despite attempts to enhance cellular uptake, the PNAs were also complexed with lipofectamine, a common transfection reagent, to enable successful transfection via endocytosis. PNAs are highly effective ligands for selectively recognizing double‐stranded RNA. Hnedzko et al.^[^ ^43^ ^]^ demonstrated that PNA sequences modified with 2‐aminopyridine (M) either alone or conjugated to lysine and arginine tripeptides can form sequence‐specific triple helices with RNA hairpins. A 9‐mer PNA modified with M was able to enter HEK293 cells at low micromolar concentrations, even without the addition of CPPs. Notably, the cellular uptake was further enhanced when the number of M‐modified nucleobases was increased in a 12‐mer PNA, marking the first reported case of a heterocyclic base modification enhancing cellular uptake. The conjugation of M‐modified PNAs with additional lysine or arginine residues gave another boost to the cellular uptake, further enhancing their delivery efficiency. The proliferation assay also showed that the PNAs were not cytotoxic at concentrations up to 10 µM. Rozners demonstrated the use of PNA oligomers with 2‐aminopyridine (M) exhibited enhanced RNA binding and improved cellular uptake. In a follow‐up study, Rozners^[^ ^44^ ^]^ utilized confocal fluorescence microscopy to assess the ability of CPPs to enhance the cellular uptake of M‐modified oligomers. The PNAs linked with Tat and octa‐arginine peptides were efficiently internalized by MCF7 cells at a concentration of 1 µM. The M‐modified PNA also showed strong uptake when the concentration was increased to 5 µM. M‐modified PNAs conjugated with peptides such as Tat, octa‐arginine, or tri‐lysine demonstrated dispersed fluorescence throughout the cytoplasm and nuclei, particularly in the nucleoli. Unlike typical mechanisms of endosomal escape, endosomolytic treatments using chloroquine and CaCl_2_ did not release the conjugates from the vesicles, indicating that they were not trapped in endosomes. The authors' hypothesis is that M‐modified PNA sequences could escape endosomes and accumulate in RNA‐rich cellular compartments, such as nucleoli, stress granules, and P‐bodies. A new PNA monomer with a cyclic tetrahydrofuran (THF) backbone, replacing ethylene diamine units, was developed by Clausse et al., forming sequences called “thyclotides” to enhance cellular uptake (Figure 6C).^[^ ^45^ ^]^ These thyclotides designed to target miRNA‐21 (overexpressed in cancers), were compared to unmodified PNAs. The cellular tests were performed with SKHEP1^[^ ^46^ ^]^ (endothelial cells isolated from a hepatic adenocarcinoma patient) and HepG2^[^ ^47^ ^]^ (hepatocellular carcinoma and hepatoblastoma cells), thyclotides reduced miRNA‐21 expression and increased the expression of the downstream targets PTEN, Cdc25a, and KRIT1, without the use of CPPs. Additionally, thyclotides showed no cytotoxicity at 200 times their active concentration. A subsequent study developed by Zhang and coworkers^[^ ^48^ ^]^ regarded the selective incorporation of THF‐derived PNAs (thyclotides), which improved both aqueous solubility and cellular uptake by pre‐organizing the PNA strand into a right‐handed helix. Thyclotides, with enhanced binding properties, exhibited slightly lower stability in duplexes compared to cpPNAs; however, they showed effective uptake into a human colorectal carcinoma cell line originated from an adult male (HCT116 cells), inhibiting miRNA‐21 expression. The optimal number of THF groups in thyclotides resulted in the highest biological activity in HCT116 cells. The sensitivity to these groups suggests that their properties can be finely tuned for maximum effect. These unique characteristics could lead to widespread use of thyclotides in various biomedical research applications. Avitabile et al.^[^ ^49^ ^]^ designed and synthesized the first sulfate PNA oligomer (S PNA) by modifying γ‐hydroxy methyl monomers. An interesting analogy emerged between the sulfate monoester and the phosphate diester of oligonucleotides, as they both carry the same charge. The group suggested several advantages such as enhanced binding affinity and improved cellular delivery for these sulfate PNAs. Cellular delivery was assessed by complexing S‐PNA with lipofectamine, comparing the complex with the standard PNA oligomer in *SKBR3* cells^[^ ^50^ ^]^ (a human breast cancer cell line known to overexpress the HER2 gene product). The data revealed that S PNA efficiently crossed the membrane, unlike the standard PNA oligomer, and reached the nucleus, possibly due to the negative charge of the sulfate group, which facilitated binding with cationic lipids. The study demonstrates promising antigene activity for S PNA as a highly selective DNA‐binding molecule.

### 2.4 Peptide‐Based Delivery Systems

One of the first delivery systems, employed to date for the delivery of PNA oligomers, is CPPs.^[^ ^51^ ^]^ CPPs are short peptides (less than 30 residues) with the ability to cross biological membranes in an energy‐dependent or energy‐independent manner.^[^ ^52^ ^]^ They have the notable ability to transport various cargos inside cells with limited toxicity. CPPs can be classified in different ways: a) on the basis of their binding properties (primary amphipathic, secondary amphipathic, and nonamphipathic); b) on the basis of their nature as protein‐derived or chimeric CPPs or completely synthetic peptides such as the polyarginine group.^[^ ^53^ ^]^ In Table 1, 2, the CPP used to deliver PNAs and the most recent advances in this field are summarized.

**Table 1:** Summary of CPP employed for PNA delivery.

Peptide	Sequence	Reference
Tat_48‐57_	GRKKRRQRRRPPQ	[54, 55]
K8	KKKKKKKK	[55]
(RXR)_4_XB	RXRRXRRXRRXRXB	[55]
Oligoarginine (R8)	R(*n*); 6 < *n* < 12	[56, 57]
Vitamine B_12_	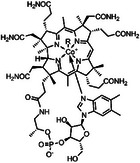	[58]
**RATH‐1**	HKTPWWRLWTKWSQPHHKRRDLPRKPE	[59]
**RATH‐2**	HKTPWWRLWTKWSQPHHKRRNLPRKPQ	[59]
**DAB (diamino butanoic acid)**	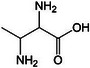	[60]
**Anoplin**	GLLKRIKTLL	[61]
**Oligolysine**	K(*n*); 1 < *n* < 6	[62]
**C9**	CCCCCCCCC	[63]

One of the first and most studied CPPs, discovered in the late 1980s, was the TAT, derived from human HIV. Despite over 30 years of study, its internalization mechanism has not been fully understood. The most widely accepted theory suggests that the translocation process occurs through electrostatic interaction and hydrogen bonds with the components of the cell membrane. In recent years the TAT peptide has also been utilized for the delivery of PNAs.^[^ ^54^ ^]^

In 2019, Barkowsky et al. explored the potential of PNAs as antisense agents to inhibit the replication of *Streptococcus pyogenes*.^[^ ^55^ ^]^ The study compared the effectiveness of anti‐gyrA PNAs targeting *gyrA*,^[^ ^64^ ^]^ an essential gene encoding a subunit in DNA *topoisomerase gyrase*, which is critical for bacterial growth.

To assess the antimicrobial impact on *S. pyogenes*, strain 591 of *S. pyogenes* M49 was exposed to 10 µM of the CPP‐anti‐gyrA PNA and incubated for 6 hours. The experiments were performed, and only three conjugates demonstrated antimicrobial efficacy: TAT‐anti‐gyrA PNA, K8‐anti‐gyrA PNA, and (RXR)_4_XB‐anti‐gyrA PNA. Additionally, all CPP‐anti‐gyrA PNAs exhibited a dose‐dependent bactericidal effect. Another group used an octo‐arginine CPP (R8) conjugated with an 8‐mer PNA in tandem (PNA1 CPP) and linked its complementary PNA to an autophagy‐inducing peptide (AIP).^[^ ^56^ ^]^ The study demonstrated that PNA can act as a useful “molecular glue” for CPP. After the delivery into cells, the complex dissociated, releasing the individual components. Due to its easy synthesis and interesting potentiality, the PNAs strategy demonstrated wide applicability in cellular delivery. In 2020, Montazersaheb et al. focused on modulating TdT gene expression using PNA's technology.^[^ ^57^ ^]^ They employed short PNA conjugates stabilized with D‐octarginine to target the TdT gene in Molt‐4 cells, which are derived from human acute lymphoblastic leukemia. A critical aspect of their approach was the CPP that contained a biologically stable D‐octarginine moiety, which significantly enhanced cellular uptake. This enhancement is essential for the effective delivery of PNAs. Pienko and coworkers showed that PNA oligomers can efficiently enter *Escherichia coli* bacteria when conjugated to vitamin B_12_, a vitamin that can be synthesized only by a few bacteria.^[^ ^58^ ^]^ The study showed that the PNA‐ B_12_ complex interacted with the bacteria in the same way as vitamin B_12_ without a carrier. Permeabilization of the B_12_–PNA occurred only after the recognition of vitamin B_12_ by BtuB, the outer‐membrane receptor of *E. coli*. Joshi et. al.^[^ ^59^ ^]^ investigated an amphipathic CPP based on the RATH peptide (TPWWRLWTKWHHKRRDLPRKPE), which was originally introduced by Bais^[^ ^65^ ^]^ for a noncovalent delivery of PNA. The RATH peptides, derived from the avian infectious bursal disease virus, typically consist of a positively charged segment and a hydrophobic tail. In their study, Joshi and colleagues explored two analogues of the RATH peptide:

RATH‐1 (HKTPWWRLWTKWSQPHHKRRDLPRKPE) and RATH‐2 (HKTPWWRLWTKWSQPHHKRRNLPRKPQ), both of which included a spacer (SQP9). The introduction of the SQP linker enhanced the interaction between tryptophan and PNA, resulting in improved cellular internalization and stronger conjugation between PNA and the peptide. Molecular interaction analysis, performed using fluorescence spectroscopy, characterized the PNA‐RATH1 and PNA‐RATH2 complexes. The study identified the RATH‐2 variant as the most efficient CPP for PNA delivery in cell culture, achieving an impressive transfection efficiency of 93%.

Recently, a series of dendrimers based on diaminobutanoic acid (DAB) have been explored as innovative carriers for PNA in the development of antibacterial agents. Nielsen et al. synthesized a DAB‐PNA conjugate as an antisense, antimicrobial, and bactericidal agent targeting the *acpP* gene in *E. coli* and *Klebsiella pneumoniae*.^[^ ^60^ ^]^ The *acpP* gene encodes an acyl carrier protein, a key component of bacterial cell metabolism. However, this protein is challenging with conventional antibiotics due to the impermeable outer lipopolysaccharide (LPS) layer that protects the bacterial cell. The DAB‐PNA conjugates demonstrated excellent stability in both mouse and human serum, highlighting DAB‐based dendrimers as promising and effective carriers for antibacterial agents. These findings suggest that DAB‐PNA conjugates could offer significant potential for in vivo applications, opening new opportunities for combating bacterial infections.

Anoplin, a novel and effective carrier peptide to deliver PNAs into bacterial cells, was introduced by Siekierska and colleagues.^[^ ^61^ ^]^ Among the tested conjugates, the anoplin–eg1–PNA conjugate exhibited significant antibacterial activity against *Escherichia coli* and *Salmonella Typhimurium* strains, with minimal inhibitory concentrations (MICs) in the 2–4 µM range. Anoplin's ability to effectively facilitate PNA uptake and exert an antisense effect on essential bacterial genes. The findings highlight anoplin as an alternative to commonly used carriers like (KFF)₃K, particularly in combating Gram‐negative bacteria, where PNA delivery remains a key challenge.

Adenoviruses have recently been used as effective transport systems for delivering drugs to suppress tumors.^[^ ^62^ ^]^ Falanga et al.^[^ ^66^ ^]^ reported that oncolytic adenoviral vectors (OAds) can target tumor cells and deliver a PNA quadruplex, able to target oncogene promotors and reduce the expression of anti‐apoptotic Bcl‐2, a protein found in various cancers.^[^ ^67^ ^]^ To improve the attraction between the anionic surface of OAd and PNA, the PNA backbone was modified by adding positively charged lysines (from 1 to 6 K), called PNA6K. The results evidenced that OAds took at least 3 days to enter cells, replicate, and produce the cytotoxic effect. The treatment with PNA6K for 5 days led to the downregulation of Bcl‐2 oncogene.

A cyclic peptide (C9) carrier was conjugated to antimiRNA‐155a sequence based on PNA, specifically designed to target mature miRNA‐155, a well‐known oncogenic miRNA.^[^ ^63^ ^]^ The antimiRNA‐155 oligomer was labeled with FITC and then tracked during its uptake into glioblastoma cells (U87MG) following the incubation at a low concentration (0.5 µM). The study revealed a 20‐fold increase in efficiency for C9 – anti‐miRNA‐155 PNA‐FITC conjugate compared to other CPP systems, such as TAT or R9. This novel class of CPPs demonstrated superior cellular delivery compared to linear CPP‐PNA conjugates, highlighting a new approach for enhancing the cellular transport of PNA.

Papi et al. investigated a new method involving the treatment of bronchial epithelial cells, specifically Calu‐3 cells, with PNA sequences targeting two miRNAs: miRNA‐145–5p and miRNA‐101–3p.^[^ ^68^ ^]^ The goal of this treatment was to synergistically increase the expression of the Cystic Fibrosis Transmembrane Conductance Regulator (*CFTR*) Gene. The combined treatment with PNAs targeting both miRNA‐145–5p and miRNA‐101–3p showed a greater effect in enhancing CFTR gene expression compared to treating with each PNA separately. Furthermore, the combined treatments also minimized the therapeutic impact on CFTR expression. Despite their potential promise in laboratory settings, CPPs for PNA delivery face clinical challenges due to high therapeutic doses, toxicity, off‐target effects, and issues with endosomal entrapment and release. Many CPPs, especially those rich in basic amino acids like arginine or lysine, can cause significant cellular damage at elevated concentrations. As a result, alternative PNA delivery methods with fewer covalent modifications, such as nanoparticles, liposomes, etc. have been explored to aid cellular entry.

## 3 Vanguard Nanotechnologies

### 3.1 Nanoparticles

**Figure 7:**
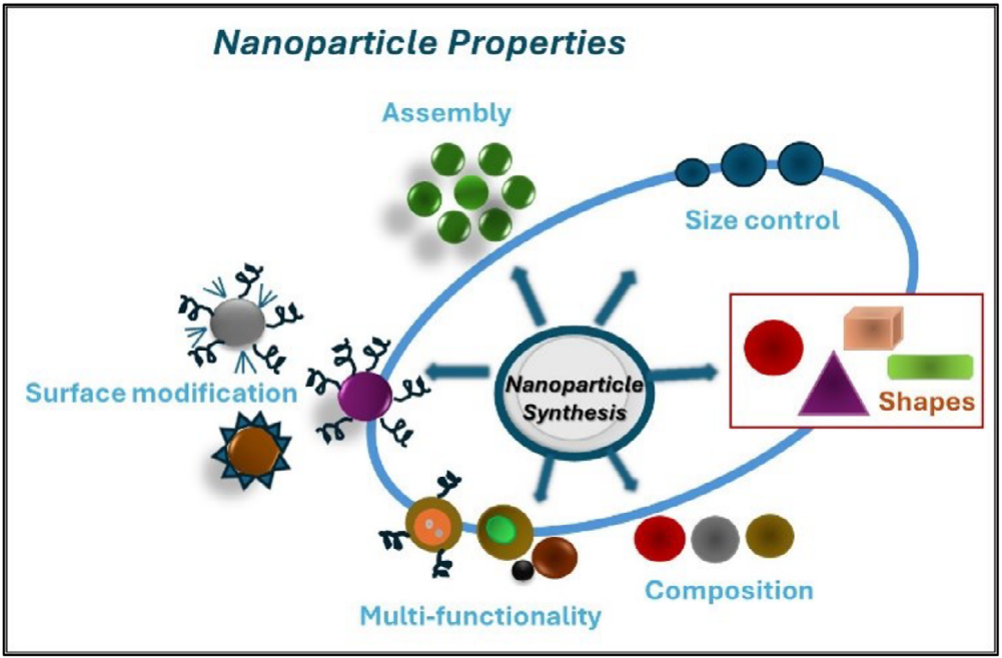
Nanoparticle properties.

Nanotechnology is a set of technologies that relies on the manipulation of matter at atomic, molecular, and supramolecular levels, involving the design, manufacture, characterization, and application of different nanoscale materials in several areas and providing novel technological advances mainly in the field of medicine (so‐called nanomedicine). Nanoparticles (NPs) are defined as particulate dispersions or solid particles with a size range of 1–300 nm and a composition of various materials with regular (tubular, spherical, or filamentous) or irregular shapes.^[^ ^69^ ^]^ Due to both quantum effects and an increased surface area per unit of mass, NPs possess peculiar properties, which confer to them greater chemical reactivity, greater resistance and electrical conductivity, and more enhanced biological activity (Figure 7).^[^ ^70^ ^]^

NPs exhibit diverse characteristics such as strength, flexibility, and durability, which intrigue disciplines like medicine, biology, and engineering.^[^ ^71^ ^]^ NPs can be tailored for multifunctional applications by engineering their surface or core, enabling their use in chemotherapy, bioimaging, and drug delivery systems.^[^ ^72^, ^73^ ^]^ They can be organic or inorganic, with examples including quantum dots and liposomes.^[^ ^74^, ^75^ ^]^ Additionally, NPs serve as stimuli‐responsive release systems,^[^ ^76^ ^]^ controlled by factors such as temperature and pH,^[^ ^77^, ^78^ ^]^ improving targeted drug delivery.^[^ ^79^, ^80^, ^81^ ^]^ Utilizing NPs in drug delivery addresses challenges associated with conventional methods, offering advantages such as improved efficacy and reduced toxicity. Overall, NPs hold immense potential in pharmacological and biotechnological applications, facilitating efficient drug delivery while overcoming obstacles like specific distribution and low bioavailability.

**Figure 8:**
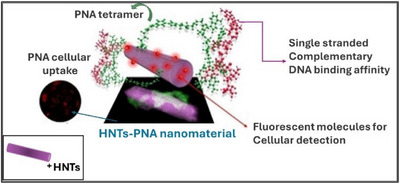
HNTs‐based PNA‐delivery system.

Malik et al. engineered PNA amphiphiles using chemically modified γPNA (8 mer in length) which consisted of hydrophilic diethylene glycol units at the gamma position and a hydrophobic moiety in the form of lauric acid (C12) covalently linked to it.^[^ ^82^ ^]^ The authors selected a γPNA sequence complementary to the seed region of oncomiR‐155, enabling it to self‐assemble into spherical vesicles. For comparison, they also investigated the same amphiphilic γPNA formulated as a polymer via ethanol injection‐based protocols. Their findings demonstrated that amphiphilic γPNAs can be successfully formulated into nanoassemblies, exhibiting superior cellular transfection and functional activity in vitro. In recent years, halloysite nanotubes (HNTs) have been developed for drug delivery applications. Halloysite can penetrate cell membranes and enhance the solubility of drugs in biological media. Massaro et al. reported the first example of a nanocarrier, based on halloysite and labeled with fluorescent switchable halochromic oxazine molecules, for the delivery of a single‐stranded PNA tetramer into living cells. The PNA tetramer was covalently attached to the halloysite, while the fluorescent probe interacted with nanotubes. The delivery of HNTs‐PNA was assessed in three different cell lines: *hTERT* (epithelial cells), *MCF‐7* (breast cancer), and *HL‐60R* (multidrug‐resistant acute myeloid leukaemia).^[^ ^83^ ^]^ In all cases, HNTs can efficiently transport PNA inside cells, localizing in the perinuclear region. Furthermore, the binding ability of HNTs‐PNA conjugate was tested using a complementary single‐stranded DNA and was found to be very effective and specific. HNTs represent highly efficient nanocarriers capable of simultaneously co‐delivering multiple active species (Figure 8).

Riela et al. proposed a study aimed to explore the potential of halloysite for applications in nanomedicine, specifically as a carrier for delivering active substances.^[^ ^84^ ^]^ Three differently charged PNA tetramers were investigated for their interaction with halloysite. The PNAs were synthesized and loaded onto HNTs, and the resulting nanomaterials were characterized using various techniques. The nanomaterials were analyzed, using FT‐IR spectroscopy and thermogravimetric analyses, to determine the loading percentage of each PNA into HNTs. DLS and z‐potential measurements revealed variations in aqueous diffusion features and surface charges among the three nanomaterials, indicating interactions between different HNT surfaces and the PNA tetramers. The study found that the interaction between charged PNAs and HNTs led to changes in nanomaterial properties, such as size and surface charge. Kinetic release experiments revealed that release mechanisms varied depending on the PNA charge. Theoretical calculations supported experimental findings, showing adsorption of PNAs onto halloysite surfaces through hydrogen bonds and electrostatic interactions. The results indicate that PNA tetramers interact differently with HNT surfaces, depending on their charge, and influence their release in media simulating physiological conditions. Overall, the study demonstrates the potential for combining PNAs with halloysite as a cost‐effective and biocompatible carrier, opening avenues for future clinical applications in treating various pathologies. On the same topic, Falanga et al. explored the use of HNTs for delivering PNA oligomers targeting the neuroglobin gene.^[^ ^85^ ^]^ Two PNAs with different charges were synthesized and loaded onto HNTs, targeting either the external surface or the internal lumen. Characterization confirmed the presence of PNAs on the HNTs and revealed selective interactions. The release kinetics of the PNAs were correlated with their interaction surfaces on the HNTs. Cellular uptake of the PNAs increased significantly when loaded onto HNTs, enabling penetration into both the cytoplasmic and nuclear regions. The inhibition of neuroglobin expression demonstrated the therapeutic potential of HNTs/PNA nanomaterials for gene silencing therapy. This study represents significant progress in utilizing HNTs as delivery systems for PNAs, offering promise for gene therapy applications. Future research aims to develop fluorescent carriers based on halloysite for the co‐delivery of PNAs and other therapeutic agents, with potential applications in treating solid tumors through oral, topical, or local administration.

#### 3.1.1 Gold Nanoparticles

**Figure 9:**
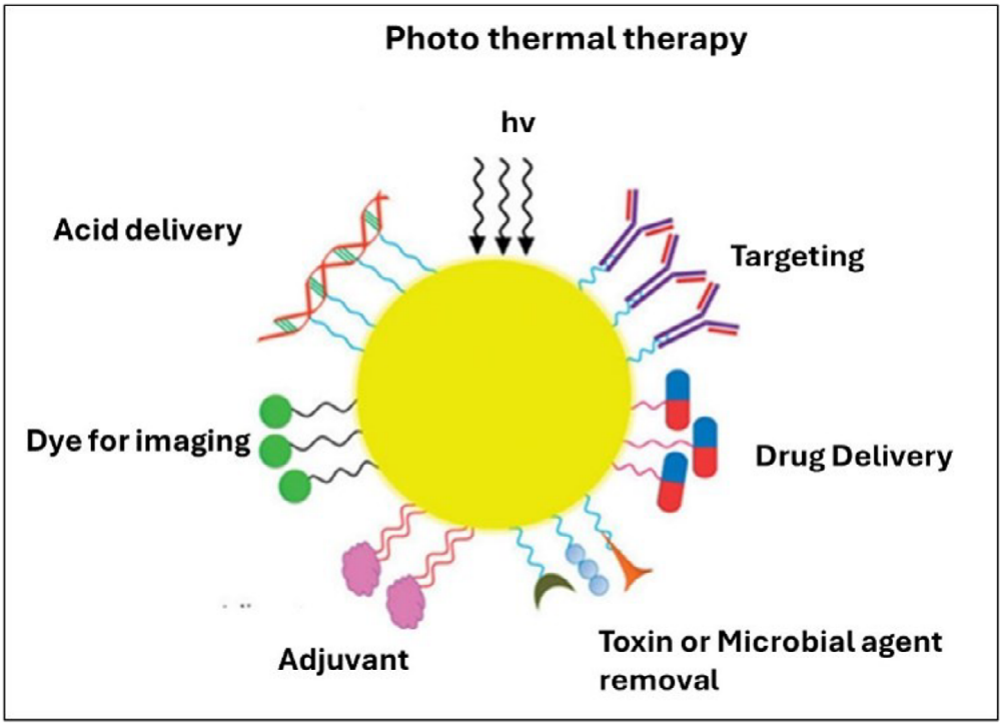
Various applications of gold nanoparticles.

The use of gold nanoparticles (Au NPs) has been widespread in the biomedical field.^[^ ^86^ ^]^ These nanoparticles can be easily synthesized in sizes ranging from 1 nm to 100 nm, with their electronic and optical properties depending on their size. Additionally, their negative charge makes them suitable for functionalization with various molecules, including drugs, genes, and target ligands (Figure 9).^[^ ^87^ ^]^

Finally, they are biocompatible, do not cause acute or adverse toxicity, and represent ideal candidates for drug delivery as well as for other biomedical applications such as biosensing and molecular imaging.^[^ ^88^, ^89^ ^]^ Despite the great potential of PNA‐based AuNPs, only a few examples in the literature report the direct conjugation of PNA with AuNPs, primarily due to the strong interaction between gold and neutral PNAs.

Nucleobases exhibit a strong tendency to directly interact with the gold surface, significantly reducing PNA layer formation and loading. To address this limitation, Anstaett et al. reported a novel conjugation technique involving a covalent linkage through a thiol‐gold bond, enabling the development of highly stable PNA‐functionalized AuNPs.^[^ ^90^ ^]^ They functionalized the PNA oligomers with a terminal thiol group, a cysteine residue, spaced with a polyethylene glycol (PEG) linker, to avoid steric hindrance with the nanoparticle surface. Ghaffari et al. employed gold nanoparticles as delivery systems for an antisense PNA oligomer targeting the 5′‐untranslated region of bovine viral diarrhea virus (BVDV).^[^ ^91^ ^]^ AuNPs protect PNA oligomers from the reticuloendothelial system (RES), facilitate their entry into the cells, and support their efficient targeting. Moreover, AuNPs enhance the pharmacokinetic properties of PNA, increasing its circulation in the bloodstream.

#### 3.1.2 Graphene Oxide Nanoparticles

**Figure 10:**
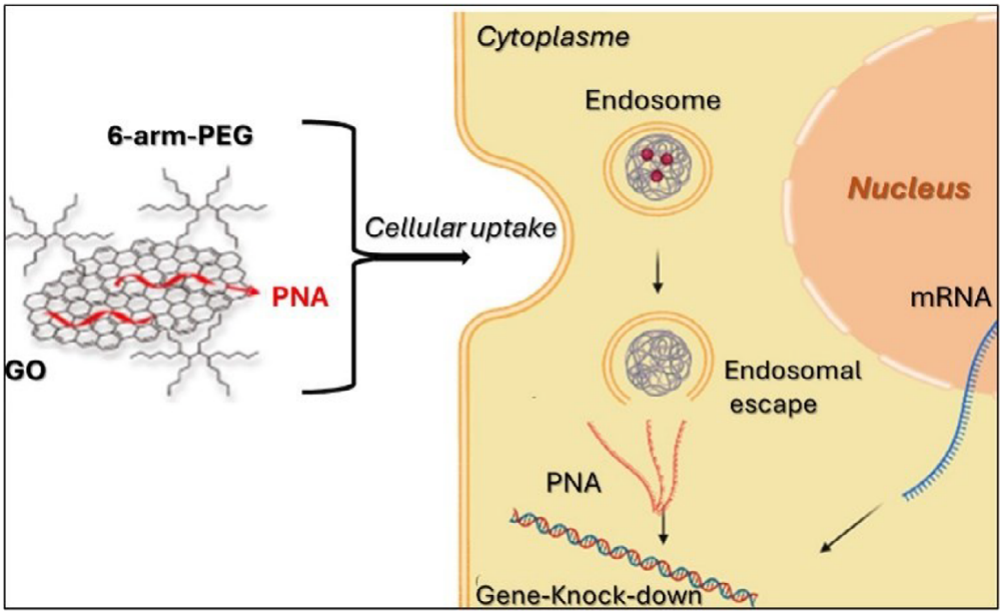
PNA‐PEGylated GO cellular uptake.

Another attractive material for advanced drug transport is graphene oxide (GO), the oxidized product of graphite with < 8 layers, which possesses a 2D planar structure, wide surface area, chemical and mechanical steadiness, and high conductivity.^[^ ^92^, ^93^ ^]^ Unfortunately, GO has several limitations including low dispersibility in culture media and significant cell cytotoxicity, and little biocompatibility. When GO was introduced into cells without proper functionalization, it decreased cell viability by activating apoptotic *caspase‐3*, inducing necrosis and autophagy through the generation of reactive oxygen species.^[^ ^94^, ^95^, ^96^ ^]^ To overcome this issue, the introduction of a polyethylene glycol (PEG) linker has been proposed as an effective strategy to enhance GO's biocompatibility. For instance, Baek et al. developed PEG‐engrafted GO as biocompatible carriers for gene delivery of antisense PNAs into cells demonstrating improved gene delivery efficiency and reduced toxicity.^[^ ^97^ ^]^ A PNA oligomer, designed to target the epidermal growth factor receptor gene (*EGFR*), was adsorbed onto PEG‐nGO through hydrophobic (π–π) interactions and efficiently delivered into *A549* lung cancer cells via endocytosis, without affecting cell viability, while exhibiting lower cytotoxicity and higher aqueous dispersibility. PNA, either in presence of complementary RNA or under low pH conditions, was completely desorbed and able to effectively knock down the expression of the target receptor gene (Figure 10).

#### 3.1.3 Silica‐Based Nanoparticles

**Figure 11:**
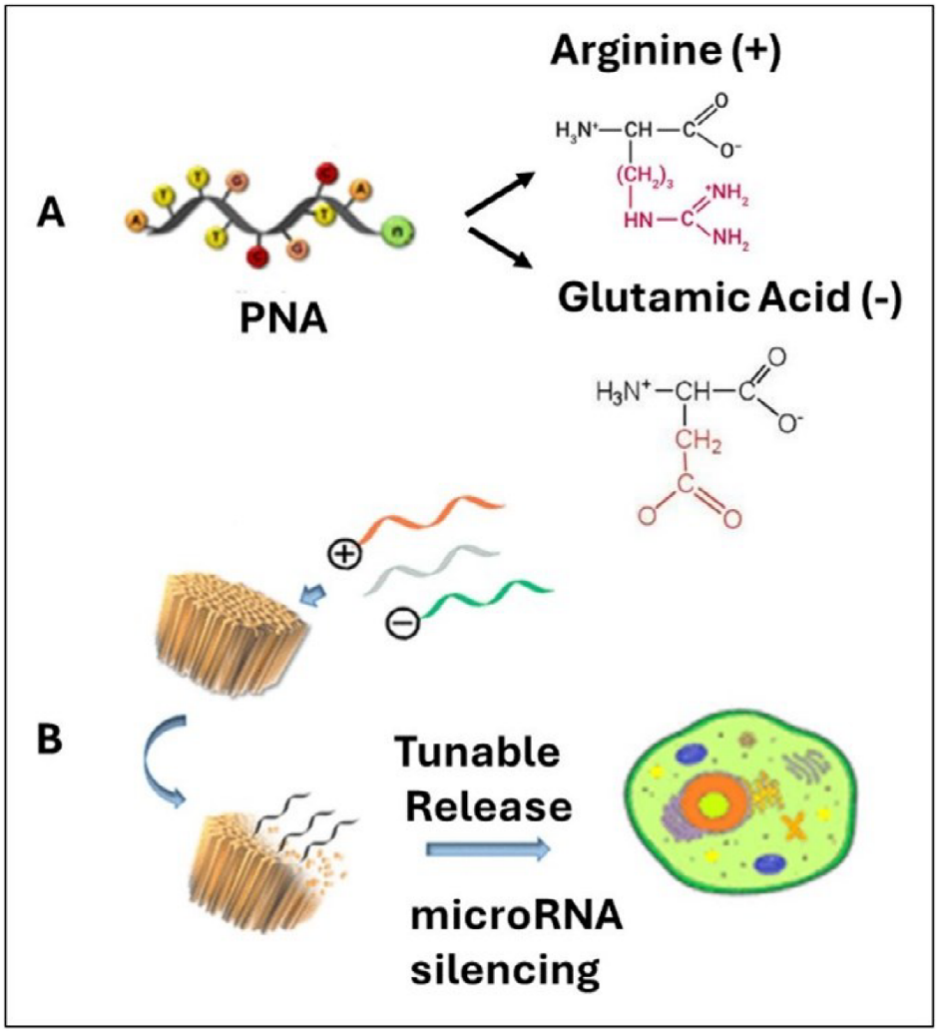
a) Depiction of a PNA oligomer linked to amino acid residues with different charges; b) Scheme of the calcium silicate trapping method for producing PNA‐loaded pSiNPs.

Silica porous‐based nanoparticles have found widespread applications as controlled drug delivery systems due to their appealing features such as a large surface area, high pore volume, abundant inner and outer surface chemistries, good biocompatibility, thermal/chemical stability, resistance to corrosion under physiological conditions, ease of synthesis, tunable properties, tunable pore size and volume. This discussion focuses on two types of silica‐based nanoparticles: zeolites and mesoporous silica nanoparticles. Among these, mesoporous silica nanoparticles (MSNPs) are currently the most extensively studied type of drug delivery system.^[^ ^98^ ^]^ Their high porosity and surface area provide significant potential for developing tailor‐made multifunctional nanocontainers. In addition to the amorphous MSNPs, crystalline silica‐based nanocarriers, such as zeolites, exhibit similar features. However, they have received significantly less attention for drug delivery or other biomedical applications.^[^ ^99^ ^]^ Neri et al. showed how to effectively load PNAs into porous silicon nanoparticles (pSiNPs), using a simple salt‐based trapping method (Figure 11).^[^ ^99^ ^]^ Researchers were able to optimize the loading of PNAs into pSiNPs and control the release rate by adjusting the net charge of the PNAs. The flexibility of PNA chemistry allows both positively and negatively charged PNAs. Positively charged PNAs, such as arginine‐based PNAs, can be easily incorporated into pSiNPs, reducing potentially harmful effects (Figure 11A). On the other hand, negatively charged payloads can also be encapsulated using this method, eliminating the need for potentially toxic cationic polymers (Figure 11A). In in vitro experiments, specific miRNAs related to cystic fibrosis were successfully silenced using this technique, indicating its potential for therapy (Figure 11B).

**Figure 12:**
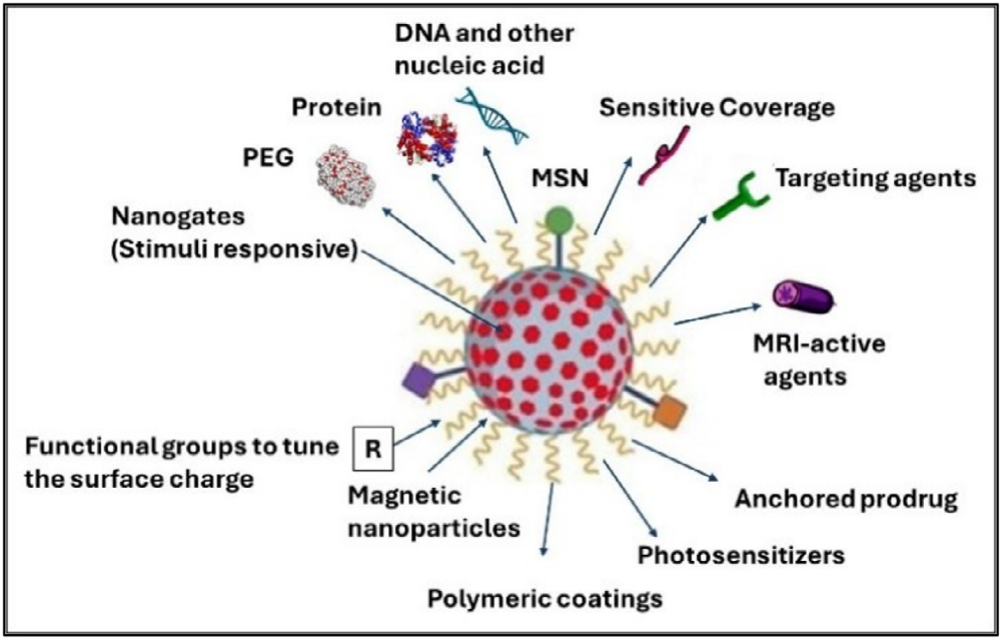
Multifunctional mesoporous silica nanoparticles.

Further refinement, including nanoparticle customization and optimization of PNA payloads, could expand the therapeutic uses of PNA‐pSiNP complexes in precision medicine. Mesoporous Silica nanoparticles: Mesoporous silica nanoparticles^[^ ^100^ ^]^ (MSNs) represent an innovative inorganic platform for biomedical and therapeutical applications. Thanks to their ordered porous structure, MSNs offer unique structural properties such as precise control over drug loading and favorable release kinetics. MSNs present two functional surfaces, the cylindrical pore surface and the exterior particle surface, that can independently control drug loading and release. Additionally, the exterior surface can be conjugated with selective ligands to enable efficient drug targeting. (Figure 12). As they have good biocompatibility and very low toxicity, they represent a good delivery system for PNA oligomers. Xang et al. employed a PNA‐based MSNP delivery system to silence B‐cell lymphoma 2 (Bcl‐2) protein expression in vitro.^[^ ^101^ ^]^ The antisense PNA, covalently linked to MSNP through a disulfide bond, entered the *HeLa cells* via *endocytosis* and selectively reached cancer cells through a redox‐controlled release mechanism, inducing an efficient silencing of Bcl‐2 protein expression. Bertucci et al. employed mesoporous silica nanoparticles loaded with the anti‐cancer drug temozolomide (TMZ) and coated with a polyarginine PNA to inhibit miRNA‐221 function and induce apoptosis of cancer cells.^[^ ^102^ ^]^ The authors reported, for the first time, the concomitant delivery of an antimiRNA‐221‐PNA‐octa‐arginine conjugate (R8‐PNA‐221) and temozolomide to drug‐resistant glioma cells, demonstrating enhanced biological effects resulting from the combined action of both agents. One major drawback of porous silica nanoparticles is the high density of surface silanol groups interacting with the surface of the phospholipids of the red blood cell membranes and resulting in hemolysis.

Another disadvantage is related to metabolic changes induced by porous silica nanoparticles leading to melanoma promotion. To prevent these negative effects, modifications of traditional MSNP have been adopted as decoration with PEG or some functional agents.^[^ ^103^ ^]^

Zeolites: Zeolites are microporous crystalline aluminosilicate compounds composed of SiO_4_ and AlO_4_ tetrahedra, in which the building blocks are arranged in a regular way, resulting in an extended uniform network of channels and pores on a nano‐ and subnanometer scale referred to as micropores.^[^ ^104^ ^]^

In particular, Zeolite‐L nanoparticles, crystalline porous particles with dimensions of tens of nanometers and a 1D channel system able to host and deliver many drug molecules, showed very promising peculiarities for in vitro and in vivo applications.

Bertucci et al. published a study in which they used zeolite‐L crystal nanocarriers to deliver a PNA oligomer that is fully complementary to a DNA sequence associated with human cystic fibrosis disease.^[^ ^105^ ^]^ This work introduces the design of smart nanomaterials with potential biomedical applications, such as bioimaging and drug delivery. The study focuses on utilizing zeolite nanocrystals as multifunctional nanocarriers to simultaneously deliver PNA and organic molecules into *HeLa cells*. Zeolite‐L nanocrystals are functionalized with PNA probes on the surface and filled with fluorescent guest molecules in the channel system. Coating the system with a thin layer of biodegradable poly‐L‐lysine significantly enhances cellular uptake. The delivery of a model drug molecule, DAPI, inserted into zeolite pores, was demonstrated in cells, indicating the multifunctional ability of the system. The study suggests that using zeolite nanosystems to deliver PNA probes targeting specific RNA sequences in living cells could offer new possibilities for theranostic and gene therapy applications. These results open new perspectives for the delivery of PNAs. By using this zeolite nanosystem to carry PNA probes designed to target specific RNA sequences of interest in living cells, new opportunities for theranostic and gene therapy applications can be explored.

#### 3.1.4 PLGA Nanoparticles

Poly(lactic‐co‐glycolic) acid (PLGA) is a widely used biodegradable polymer in drug delivery systems and medical devices^[^ ^106^ ^]^ for various clinical applications.^[^ ^107^ ^]^ One of the key advantages of PLGA is its ability to undergo hydrolysis within cells, breaking down into endogenous, nontoxic metabolites, lactic acid and glycolic acid, thereby enhancing its biocompatibility for in vivo use.^[^ ^108^ ^]^

**Figure 13:**
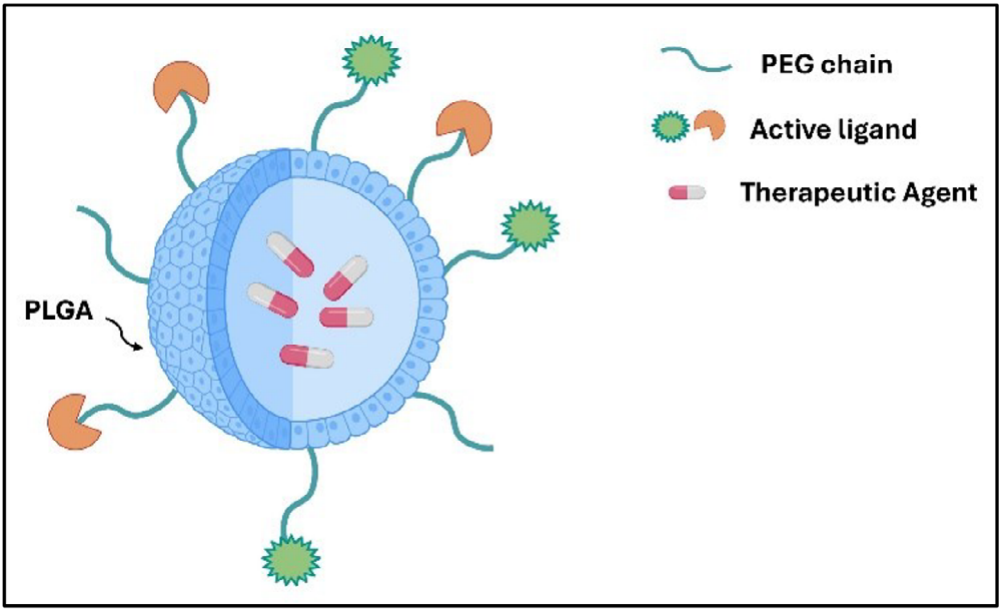
Scheme of a molecularly targeted stealth PLGA‐NP.

PLGA‐based delivery systems have recently been developed and applied in diverse gene therapy applications, including gene editing and gene targeting (antisense) strategies. For example, nanoparticles based on PLGA have been employed for site‐specific gene editing in vitro and in vivo in relevant human cell types.^[^ ^109^, ^110^ ^]^ To prevent nanoparticle elimination by RES and to prolong their half‐life in blood circulation, PLGA nanoparticles have been decorated with chemical functionalities such as PEG or peptides. These strategies make them “stealth” and ensure sufficient circulation time to deliver the cargo to the target (Figure 13).

Babar et al. recently demonstrated that PNA‐antimiRNA‐155, encapsulated in PLGA nanoparticles, can be employed to inhibit miR‐155 functions and decrease its level in B‐cell lymphomas.^[^ ^111^ ^]^ This approach effectively silenced miRNA‐155, an oncogenic microRNA involved in several cancers, showcasing the therapeutic potential of PLGA‐based nanoparticles for miRNA modulation. However, cellular uptake efficiency may be limited without an active targeting system. To improve the pharmacokinetic properties of PNA antimiRNA‐155, a CPP, known as Antennapedia was attached to the surface of the nanoparticles, enabling the active targeted delivery of the cargo without compromising the loading capability of the nanosystems. The use of CPPs can sometimes lead to off‐target effects and may increase systemic toxicity, which requires careful dose optimization. PNAs entrapped in PLGA‐based nanoparticles have also been used for targeting messenger RNA (mRNA). Bahal et al. employed a PLGA‐based nanoparticle system to deliver antisense γPNA oligomers targeting the chemokine receptor 5 (CCR5) mRNA in the THP1 cell line.^[^ ^112^ ^]^ This approach effectively reduced CCR5 expression in an antisense manner, demonstrating the versatility of the platform for mRNA silencing beyond miRNAs. Despite the success in vitro, in vivo stability and delivery to target tissues remain significant challenges that need further optimization. Gene editing can also be achieved with PNA‐based PLGA nanoparticles. In a study carried out by Nicole et al., a triplex‐forming PNA and a donor DNA were delivered using PLGA‐based nanoparticles for site‐specific genome editing of human hematopoietic cells in vivo.^[^ ^113^ ^]^ Triplex‐forming PNAs facilitate the recombination of 50–60 bp donor DNA fragments with genomic DNA to make gene modifications. These PNA molecules form a PNA‐DNA‐PNA triplex, activate the cell's DNA repair machinery, promote recombination with donor DNAs, and cause changes in targeted genes. This approach enables precise, site‐specific editing without relying on nuclease‐based systems like CRISPR, thus reducing the risk of off‐target effects. However, PLGA nanoparticles alone exhibit limited loading capacity for high amounts of PNA oligomers, which may hinder therapeutic efficacy. To address this issue, a combination of PLGA with cationic polymers was investigated. The most effective approach was a blend of PLGA and poly(beta‐amino‐esters) (PBAE), which demonstrated superior efficiency and reduced toxicity compared to PLGA nanoparticles alone when used for targeting genomic DNA or in antisense approaches.^[^ ^114^ ^]^ The PLGA/PBAE blend enhances PNA loading, transfection efficiency, and reduces cytotoxicity. Despite improvements, challenges remain in achieving high‐level, tissue‐specific delivery in clinical settings. Additionally, grafting the surface of the PLGA/PBAE system with NLS enhances the internalization of triplex PNAs and donor DNA into airway cells, achieving gene editing efficiencies of up to 0.6% in alveolar epithelial cells and 0.3% in lung macrophages. Functionalization with NLS significantly boosts nuclear delivery, improving editing efficiency in difficulttotransfect cells. McNeer et al. applied this approach for Cystic Fibrosis (CF) therapy, employing nanoparticles to deliver triplex‐forming PNA molecules for gene editing of *F508* gene deletion.^[^ ^115^ ^]^ They encapsulated tail‐clamp PNAs and donor DNA molecules in PLGA/PBAE‐based nanoparticles coated with Model Peptide carrier peptide (MPG) to correct the *F508* in CFTR mutation both in vitro in human bronchial epithelial (HBE) cells and in vivo in a CF murine model. Although promising, translation to human clinical settings may be hindered by the complexity of nanoparticle design and limited editing frequencies. Finally, Glazer and coworkers reported an example of successful delivery, developing a γ‐modified tail‐clamp PNA (ser‐γ‐PNA), which bears a hydroxymethyl side chain that preserves helical preorganization while simplifying synthesis.^[^ ^115^ ^]^ Delivered via PLGA nanoparticles, ser‐γ‐PNA displays superior strand invasion capabilities and higher binding affinity to double‐stranded DNA compared to unmodified PNAs. In primary mouse bone marrow cells, ser‐γ‐PNA induces significantly elevated gene editing frequencies, demonstrating its potential as a highly effective and accessible tool for nanoparticle‐mediated genome editing applications. The γ‐modification greatly enhances binding efficiency and editing performance, offering a more potent and synthetically accessible alternative to standard PNAs. Despite improved molecular performance, the overall nanoparticle formulation still suffers from limited payload capacity, which may restrict clinical scalability.

Although these PLGA‐based nanoparticles have shown considerable promise, their synthesis does not currently yield nanoparticles with sufficiently high PNA payloads required for clinical efficacy. For these reasons, other nanoparticle formulations have been employed for PNA internalization and delivery.

### 3.2 Liposomes

**Figure 14:**
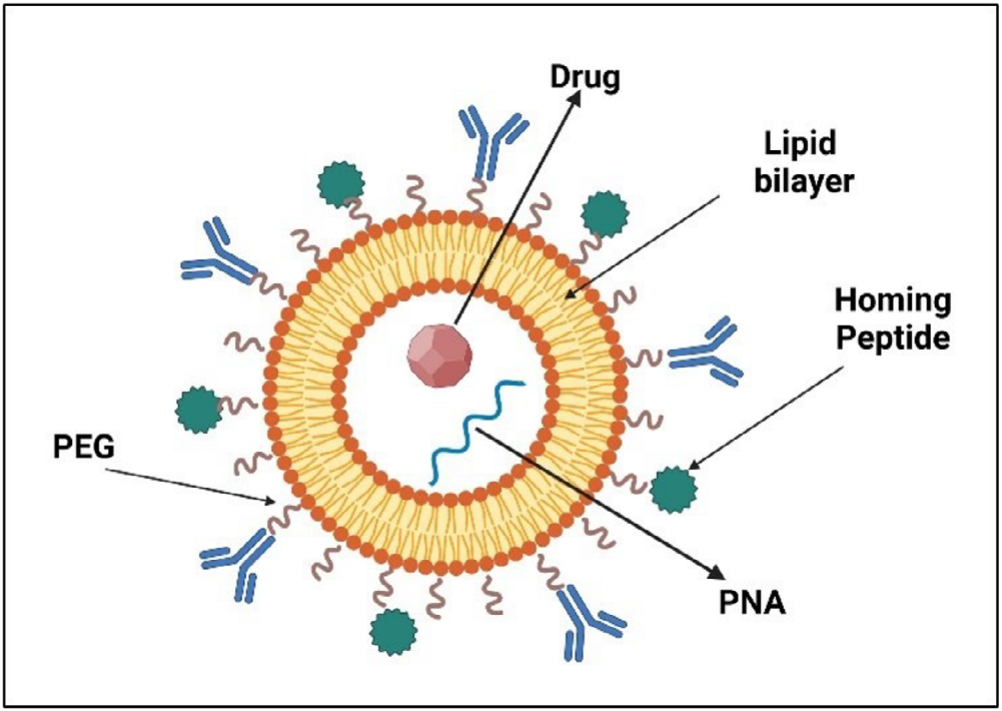
Liposomes for drug delivery.

Liposomes are among the most popular drug delivery systems introduced in recent decades. These vesicles are composed of a double phospholipid layer and can incorporate lipophilic molecules as well as encapsulating hydrophilic compounds within their aqueous core (Figure 14).^[^ ^116^ ^]^ Their large aqueous cavity allows them to encapsulate and transport hydrophilic drugs or macromolecules, including nucleic acids, peptides, and imaging agents. Lipophilic drugs are incorporated within the lipid phase of the phospholipid bilayer, allowing for delivery without the need for surfactants, adjuvants, or co‐solvents, which may otherwise cause cytotoxic effects.

Liposomes can be classified based on their surface charge as neutral, cationic, or anionic. They can also be categorized by structure and diameter into ultra‐small, small, medium, and large liposomes or further distinguished as unilamellar, multilamellar, or multivesicular vesicles. A wide range of physicochemical and biophysical properties can be fine‐tuned to control their biological functions and exploit their unique pharmaceutical characteristics, such as good biocompatibility and high self‐assembly capacity. Moreover, liposomes can function as “masking systems,” forming compartments that physically isolate the encapsulated drug from the surrounding environment. This protects the drug from chemical or enzymatic degradation and prevents dilution in the bloodstream until it reaches the target tissue.^[^ ^117^ ^]^

Besides classic liposomes, PEG‐coated liposomes, also known as PEGylated liposomes,^[^ ^118^ ^]^ can be effectively used. When PEG is attached to liposomes, it forms an external hydrophilic layer that reduces opsonization, thereby preventing rapid uptake by the RES. Due to these remarkable properties, liposomes have also been used for the delivery and selective release of antisense PNA oligomers. However, only a few examples of liposomal formulations for the delivery of PNAs have been reported in the literature.

Avitabile et al. successfully delivered a standard PNA oligomer, composed exclusively of commercially available PNA monomers complementary to miRNA‐210a, a miRNA implicated in numerous pathologies, including thalassemia. The PNA was encapsulated in a liposome with an optimized formulation, demonstrating the potential of liposomal systems for targeted delivery.^[^ ^119^ ^]^ A PNA oligomer, successfully loaded into a PEGylated liposome composition based on zwitterionic egg phosphatidylcholine, cholesterol, and 1,2‐distearoyl‐sn‐glycero‐3‐phosphoethanolamine‐N‐[carbonylmethoxy (polyethylene glycol) 2000] (egg PC/Chol/DSPE‐PEG2000, 47/47/6 molar % ratio), was rapidly and efficiently delivered into K562 cells, resulting in a reduction of miRNA‐210 expression without inducing toxicity. Experimental data showed that LIPO‐antimiRNA‐210 was able to enter cells in a manner comparable to antimiRNA‐210 conjugated to the polyarginine carrier peptide R8 (used as a positive control).

This formulation led to a faster and higher cellular uptake of PNA oligomers compared to PNA‐Arg8 conjugates. Notably, it marked an important milestone as the first example of delivering a “naked” PNA oligomer without the need for costly chemical modifications or covalent conjugations. Finally, by varying the liposomal formulation, it is possible to modulate and enhance the encapsulation efficiency and retention of PNA oligomers within liposomes.

Ringhieri et al. found that PEGylated DOPG liposomes (1,2‐dioleoyl‐sn‐glycero‐3‐phosphocholine, cholesterol/1,2‐distearoyl sn‐glycero‐3‐phosphoethanolamine‐N[carbonylmethoxy (polyethylene glycol) 2000]) with a percentage of cholesterol of 10–20% represent the most effective formulation for the loading of PNA‐antimiR‐210 (DOPG/Chol/DSPE PEG2000, 74/20/6, or 84/10/6 molar % ratio).^[^ ^120^ ^]^ The presence of cholesterol slightly decreases the PNA loading but enhances the PNA's release in fetal serum from the liposomal formulation.

The development of small multilamellar liposomes, containing large quantities of PNA, represents a promising advancement in cancer treatment. These liposomes can encapsulate significant amounts of PNAs and be engineered to deliver them specifically to breast cancer cells while minimizing off‐target effects on healthy cells. The multilamellar structure provides stability and protection to the encapsulated PNAs, preserving their integrity and functionality until they reach the targeted cancer cells. Once the liposomes containing PNAs selectively accumulate in breast cancer cells, the PNAs can interfere with crucial cellular processes by binding to specific genetic sequences. This interference can inhibit the expression of genes essential for cancer cell survival or activate pathways promoting cell death. This targeted approach enables the destruction of cancer cells while minimizing damage to healthy tissues, thereby reducing the adverse effects commonly associated with conventional chemotherapy. For example, Proshinka et al. developed new small multilamellar liposomes (SMLs), able to encapsulate and deliver large amounts of PNA.^[^ ^121^ ^]^ These liposomes were composed of a mixture of natural phospholipids and short (6–22 bases), cytosine‐rich PNA. Under mildly acidic conditions, small multilamellar vesicles measuring 60–90 nm in diameter were formed. Protonation of cytosine rendered PNA molecules positively charged, enabling electrostatic binding to negatively charged phospholipid membranes. This liposomal formulation was evaluated for its activity against the human epidermal growth factor receptor 2 (HER2), which is overexpressed in human breast cancer. The conjugate was found to enter HER2‐overexpressing cells through receptor‐mediated endocytosis. Once released from lysosomes, the PNA molecules aggregated in the cytoplasm and disrupted normal cellular processes, ultimately causing cell death.

### 3.3 Bicelle‐PNA Nanocomplexes

Bicelles are composed of aliphatic long‐chain phospholipids (12–18 carbon atoms), which form a planar region, and short‐chain lipids (6–8 carbon atoms) or detergents that constitute the surrounding edges. These structures are versatile tools that, depending on their size, composition, and hydration, can be utilized as nanocarriers to efficiently deliver hydrophobic drug molecules into cells. Bicelles have been shown to enhance drug delivery by 3‐ to 12‐fold compared to chemically identical liposomes.^[^ ^122^ ^]^

The size of bicelles can be fine‐tuned by adjusting the lipid/detergent ratio, while the surface charge can be modified by substituting neutral long‐chain lipids with phospholipids that have similar diacyl chain lengths but negatively charged headgroups. Bicelles can also be functionalized with PEGylated lipids to enhance their stability or grafted with amphiphilic biomolecules to facilitate studies on membrane proteins. Positively charged bicelles, while capable of transporting molecules into cells, often accumulate in large quantities and persist for extended periods, leading to significant cytotoxic effects on living cells. To mitigate this issue, negatively charged lipids have been employed.

**Figure 15:**
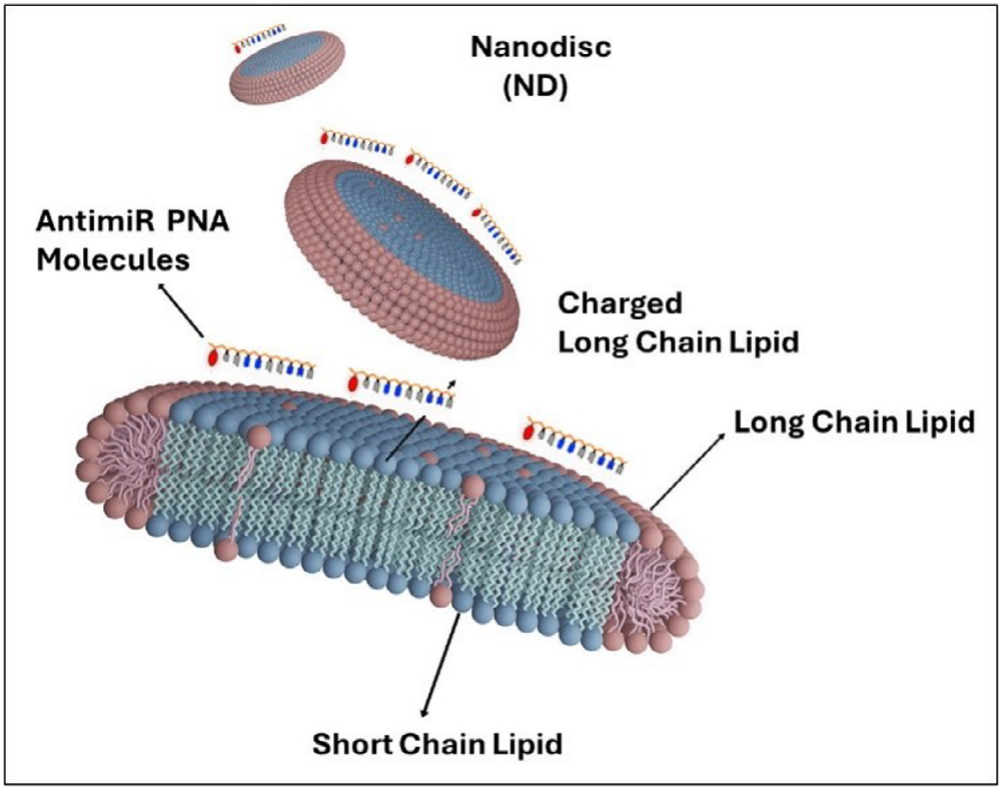
Scheme of bicelle structure.

Tahmasbi et al. investigated a discoidal nanoplatform based on bicelles for the intracellular delivery of PNAs,^[^ ^123^ ^]^ evaluating different ranges of positively and negatively charged bicelles with various PNA/lipid molar ratios. This study revealed that pegylated and negatively charged bicelles (at a ratio of 1:2500), with uniform size and discoidal shape, were highly stable and efficiently delivered PNA antimiRNA‐210 and PNA antimiRNA‐155 in HeLa cells, resulting in minimal cytotoxic effects. This led to a reduction of miRNA‐155 expression via an antisense approach. Furthermore, coating PNA‐loaded bicelles with specific peptides, antibodies, and carbohydrate units can improve accessibility to molecular targets in a highly specific manner, providing an innovative platform for PNA as therapeutic agents. A previous study by the same authors, demonstrated an improved cellular uptake of bicelles, approximately 5–10 times higher than that observed for spherical vesicles with identical chemical composition, attributed to their ability to utilize more internalization pathways compared to liposomes.^[^ ^124^ ^]^ Bicelles also offer enhanced uptake, employ diverse mechanisms for endocytosis, and exhibit faster diffusion across membranes due to their small size, making them superior candidates compared to conventional polymeric and inorganic spherical nanocarriers (Figure 15). Malik et al. engineered PNA amphiphiles by modifying gamma PNAs with hydrophilic diethylene glycol units and hydrophobic lauric acid, resulting in their self‐assembly into spherical vesicles and nano‐assemblies (NAs) using an ethanol injection protocol.^[^ ^82^ ^]^

Physicochemical and cellular uptake properties of self‐assembled PNAs and NAs were comprehensively compared, with small‐angle neutron and X‐ray scattering revealing ellipsoidal morphology in NAs, resulting in superior cellular delivery compared to spherical self‐assemblies. The functional activities of PNAs in lymphoma cells were assessed, confirming the effectiveness of PNA amphiphiles in formulating NAs for biomedical applications. This approach offers a simple method to enhance cellular transport of PNAs, potentially bypassing the need for carrier systems. The study highlights the potential of PNAs as biopolymers for designing functional nanostructures with applications in gene targeting and beyond, indicating avenues for treating cancer and metabolic disorders. Further investigation into the stability, safety, and efficacy of amphiphilic γPNAs in vivo is warranted, indicating  promising role of PNAs in future biomedical applications as enzymatically stable and nonimmunogenic biomaterials.

### 3.4 Calixarene‐PNA‐Based Delivery

Supramolecular approaches have been widely employed to develop drug delivery systems. Different synthetic macrocycles such as calixarenes or cyclodextrins have attracted attention among researchers in various fields, especially in therapeutic applications.^[^ ^125^ ^]^

**Figure 16:**
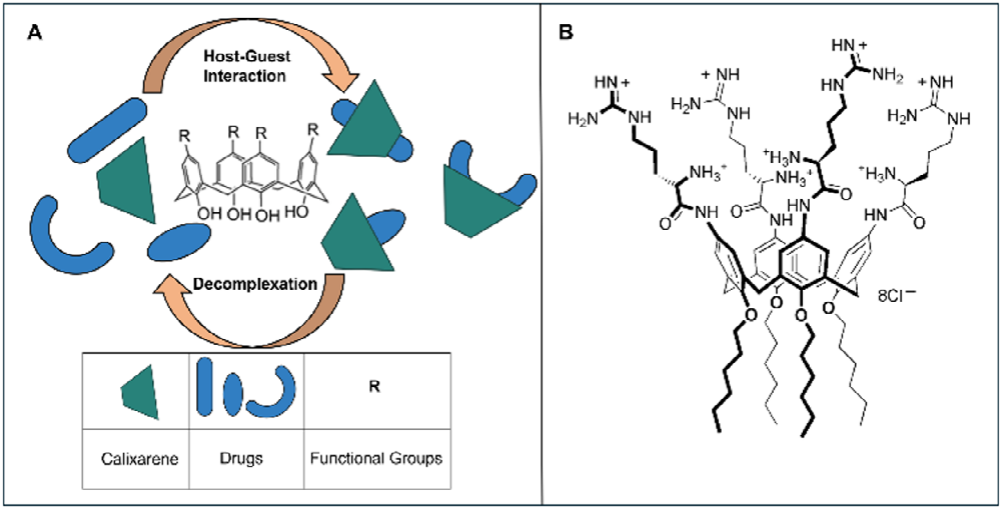
a) Calixarene‐derived inclusion complexes; b) Calixarene system employed for PNA delivery.

Calixarenes are cone‐shaped supramolecular macrocycles that can encapsulate drug molecules in their hydrophobic, electron‐rich cavities, facilitating their delivery to cells via an inclusion mechanism. These macrocycles are composed of phenolic rings connected by a methylene bridge (Figure 16A). Depending on their specific functionalization, calixarenes can exhibit good biocompatibility and noncytotoxicity.^[^ ^126^ ^]^

For these reasons, calixarene‐based systems may represent an effective tool to improve drug delivery and therapeutic efficacy as well as to minimize side effects. These systems can assemble supramolecular inclusion complexes, enabling cargo molecules to occupy the cavities through hydrophobic interactions, π‐π stacking, or charge‐transfer interactions depending on the nature of the functional groups on the calixarene moieties. This enables efficient transportation of the cargo to the target, followed by their release through a disaggregation mechanism of the complexes under chemically controlled conditions such as acidic or alkaline environments. Thus, it is possible to release the drug in a highly selective manner, allowing active targeting exclusively on the site of interest.

Starting from this observation, Gasparello et al. showed that arginine‐functionalized calix[4]arene systems can also represent a suitable tool to deliver PNA in cells (Figure 16B).^[^ ^127^ ^]^ Calixarenes, due to their uniqueness, are capable of encapsulating PNAs within their hydrophobic cavities. A neutral antimiRNA‐221–3p PNA encapsulated within a calixarene‐based system was efficiently carried into human glioma *U251* cells and was able to knock down miRNA‐221. The cone geometry of the system created two distinct regions, one hydrophobic and the other hydrophilic, imparting an amphiphilic nature to the carrier. The presence of guanidinium groups, achieved through derivatization of the supramolecular complex with four arginines, was sufficient to enable cellular uptake of PNA oligomers, avoiding lysosomal degradation and facilitating the release through a proton sponge effect.^[^ ^127^ ^]^ The formation of these complexes, involving the uncharged nature of PNAs, is primarily facilitated by π‐π stacking between the aromatic rings of the nucleobases of PNA and the phenolic groups of the vector. Additionally, charge interactions occur between the guanidinium groups of vectors and different nucleobases, in particular guanine. Cationic calix[4]arenes show promise for delivery of antimiRNA PNAs and miRNA mimics used for therapeutic applications.^[^ ^128^ ^]^ This delivery method has the potential to modulate gene expression, regulate cellular pathways, and provide treatments for various diseases such as cancer, viral infections, and genetic disorders. Therefore, this strategy merits further exploration as a potential alternative to conventional nanoparticle‐ and CPP‐based methods. However, challenges such as optimizing delivery efficiency, reducing cytotoxicity, and ensuring specific targeting need to be addressed. Current research focuses on improving vector properties, exploring new modifications, and enhancing delivery strategies to maximize therapeutic efficacy while minimizing side effects. All nanoparticle‐based systems discussed herein exhibit beneficial properties for PNA delivery. However, in addition to delivery efficiency, the biocompatibility and potential toxicity of these systems must also be carefully evaluated. Liposomes, while highly biocompatible, have a tendency to accumulate in tissues, which can lead to toxicity.^[^ ^129^ ^]^ In our opinion, PLGA/PBAE‐based nanoparticles represent the most promising system for PNA delivery, being able to convey PNA oligomers into the cytoplasm and nucleus of cells with no observable toxicity following multiple systemic injections in mice. All the mentioned nanoparticle delivery systems require proper functionalization for effective active targeting.

Currently, in vivo assays are needed to better understand the toxicity profiles and identify the optimal features of each system, enabling the discovery of the most suitable combination to balance toxicity and therapeutic effects. In all cases, we speculate that engineering nanocarriers decorated with targeting ligands may offer novel opportunities for diagnostic and therapeutic development.

**Table 2:** PNA delivery system advantages and disadvantages.

Carrier type	Advantages	Disadvantages
Cell‐Penetrating Peptides (CPPs)^[^ ^54^, ^55^, ^56^, ^57^, ^58^, ^59^, ^60^, ^61^, ^62^, ^63^, ^64^, ^65^, ^66^, ^67^ ^]^	Efficient cellular uptake, targeting,	Endosomal escape, limited stability, potential immunogenicity, toxicity
Gold Nanoparticles (AuNPs)^[^ ^86^, ^87^, ^88^, ^89^, ^90^, ^91^ ^]^	Easy functionalization, stable, high PNA loading capacity	Toxicity, limited biodegradability, high cost
Graphene Oxide (GO)^[^ ^92^, ^93^, ^94^, ^95^, ^96^, ^97^ ^]^	High surface area, controlled release, biocompatibility	Toxicity at high concentrations, functionalization challenges
Mesoporous Silica Nanoparticles (MSNs)^[^ ^100^, ^101^, ^102^, ^103^ ^]^	High loading capacity, controlled release, biodegradable	Potential toxicity, functionalization challenges, slow degradation
Zeolites^[^ ^104^, ^105^ ^]^	Ion exchange, stable, biocompatible	Limited functionalization, low loading capacity, potential toxicity
PLGA^[^ ^106^, ^107^, ^108^, ^109^, ^110^, ^111^, ^112^, ^113^, ^114^, ^115^ ^]^	Biodegradable, sustained release, well‐characterized	Slow release, inflammatory response, low PNA loading efficiency
Liposomes^[^ ^116^, ^117^, ^118^, ^119^, ^120^, ^121^ ^]^	Biocompatible, versatile, controlled release	Stability issues, short circulation time, costly
Bicelles^[^ ^122^, ^123^, ^124^, ^125^ ^]^	Tunable, biocompatible, improved effciency	Toxicity when positively charged
Calixarenes^[^ ^126^, ^127^, ^128^, ^129^ ^]^	Versatile encapsulation, controlled release, biocompatible	Stability issues, scalability issues

## 4 Conclusion

In conclusion, the development of peptide nucleic acid (PNA) delivery systems remains an area of active research. PNAs offer unique biochemical properties that distinguish them from other nucleic acid analogues. Due to their neutral backbone and strong affinity for complementary nucleic acid sequences, PNAs have demonstrated potential for gene silencing, antisense strategies, and diagnostic applications in preclinical studies. However, translating these features into therapeutic impact has proven challenging.

A significant limitation lies in the effective delivery of PNAs, with persistent issues related to poor cellular uptake, limited biological stability, and possible off‐target effects or toxicity. While various nanocarrier platforms, including liposomes, polymeric nanoparticles, and peptide‐based systems, have shown encouraging results in improving intracellular delivery, these technologies are still in early stages and require further refinement. Two examples of PNA in the pre‐clinical phase highlight how these applications are becoming tangible. Some optimists believe that the long‐awaited breakthroughs in PNA therapeutics may soon be within reach, as the field continues to make progress. However, a clear and balanced understanding of both the capabilities and current limitations of PNA technologies is crucial for realistically assessing their potential.

## Conflict of Interests

The authors declare no conflict of interest.

## Data Availability

The data that support the findings of this study are available from the corresponding author upon reasonable request.
